# Detection of Histoplasma DNA from Tissue Blocks by a Specific and a Broad-Range Real-Time PCR: Tools to Elucidate the Epidemiology of Histoplasmosis

**DOI:** 10.3390/jof6040319

**Published:** 2020-11-27

**Authors:** Dunja Wilmes, Ilka McCormick-Smith, Charlotte Lempp, Ursula Mayer, Arik Bernard Schulze, Dirk Theegarten, Sylvia Hartmann, Volker Rickerts

**Affiliations:** 1 Reference Laboratory for Cryptococcosis and Uncommon Invasive Fungal Infections, Division for Mycotic and Parasitic Agents and Mycobacteria, Robert Koch Institute, 13353 Berlin, Germany; McCormick-SmithI@rki.de (I.M.-S.); RickertsV@rki.de (V.R.); 2 Vet Med Labor GmbH, Division of IDEXX Laboratories, 71636 Ludwigsburg, Germany; charlotte.lempp@gmx.de (C.L.); Ursula-Mayer@idexx.com (U.M.); 3 Department of Medicine A, Hematology, Oncology and Pulmonary Medicine, University Hospital Muenster, 48149 Muenster, Germany; ArikBernard.Schulze@ukmuenster.de; 4 Institute of Pathology, University Hospital Essen, University Duisburg-Essen, 45147 Essen, Germany; Dirk.Theegarten@uk-essen.de; 5 Senckenberg Institute for Pathology, Johann Wolfgang Goethe University Frankfurt, 60323 Frankfurt am Main, Germany; S.Hartmann@em.uni-frankfurt.de

**Keywords:** *Histoplasma* qPCR, broad-range qPCR, formalin-fixed paraffin-embedded (FFPE) samples, histoplasmosis

## Abstract

Lack of sensitive diagnostic tests impairs the understanding of the epidemiology of histoplasmosis, a disease whose burden is estimated to be largely underrated. Broad-range PCRs have been applied to identify fungal agents from pathology blocks, but sensitivity is variable. In this study, we compared the results of a specific *Histoplasma* qPCR (*H*. qPCR) with the results of a broad-range qPCR (28S qPCR) on formalin-fixed, paraffin-embedded (FFPE) tissue specimens from patients with proven fungal infections (*n* = 67), histologically suggestive of histoplasmosis (*n* = 36) and other mycoses (*n* = 31). The clinical sensitivity for histoplasmosis of the *H*. qPCR and the 28S qPCR was 94% and 48.5%, respectively. Samples suggestive for other fungal infections were negative with the *H*. qPCR. The 28S qPCR did not amplify DNA of *Histoplasma* in FFPE in these samples, but could amplify DNA of *Emergomyces* (*n* = 1) and *Paracoccidioides* (*n* = 2) in three samples suggestive for histoplasmosis but negative in the *H.* qPCR. In conclusion, amplification of *Histoplasma* DNA from FFPE samples is more sensitive with the *H.* qPCR than with the 28S qPCR. However, the 28S qPCR identified DNA of other fungi in *H*. qPCR-negative samples presenting like histoplasmosis, suggesting that the combination of both assays may improve the diagnosis.

## 1. Introduction

While histoplasmosis is a reportable disease in several states of the USA, estimates of the global burden are difficult. Reports from South and Central America and the Caribbean suggest that histoplasmosis is an important acquired immune deficiency syndrome (AIDS)-defining infection and a major killer of human immunodeficiency virus (HIV)-infected patients [1,2,3,4,5,6,7,8]. The mortality rate of HIV-associated histoplasmosis is estimated to be equal to or even higher than for tuberculosis [8,9,10]. In a recent report reviewing 470 published histoplasmosis cases from Africa between 1952 and 2007, the authors concluded that the prevalence of histoplasmosis may be underestimated, as a large proportion of disseminated cases may be misdiagnosed as culture-negative tuberculosis [11].

The diagnosis of histoplasmosis is challenging due to an unspecific clinical presentation and suboptimal diagnostic tests. Cultivation of *Histoplasma* remains the gold standard for the diagnosis of histoplasmosis, but slow growth and low culture sensitivity reduce its usefulness [12]. In fact, the sensitivity of cultures may vary from 26% to 58% depending on the clinical manifestation, the net state of the immunity, and the severity of disease [13]. Antibody detection tests may be negative during the first four to eight weeks, especially in patients with impaired humoral immunity [13]. Moreover, false-positive results are possible with other invasive fungal infections [13]. Still, *Histoplasma* antigen testing could be a helpful tool in immunosuppressed patients with disseminated disease, but its availability is limited in many countries [14,15]. Serum *Aspergillus* galactomannan, which is widely available, has shown cross-reactivity with *Histoplasma* and may have some utility in detection of disseminated histoplasmosis in patients with AIDS [16]. However, its utility is limited by the lack of specificity [16].

Tissue biopsies are frequently necessary to confirm histoplasmosis, but cultures may remain negative and the demonstration of small budding yeasts clustering in phagocytes by histopathology is not pathognomonic [17]. For example, the recently described emergomycosis, or more prevalent mycoses including candidiasis, could potentially be misdiagnosed as histoplasmosis [18]. Previously, thorough microscopic examination of pathology blocks helped to document changes in the epidemiology of invasive fungal infections, including the increases of mold infections such as mucormycosis [19,20]. Pathology blocks are stored in tissue archives and may provide a valuable resource to gain insights into the epidemiology of histoplasmosis. In order to optimize the identification of fungal pathogens in pathology blocks, broad-range PCRs have been applied but are limited by DNA degradation due to fixation and contamination by fungal DNA [21,22].

In this study, we applied a qPCR specific for *Histoplasma* (*H.* qPCR) published in 2008 by Simon et al. [23] that demonstrated a high sensitivity (95.4%) and specificity (96%) using 348 fresh human samples from patients with high pretest probability for histoplasmosis. Interestingly, 11 culture-negative samples of proven histoplasmosis patients yielded positive results in the *H.* qPCR [23]. We compared the clinical sensitivity and specificity [24] of this *H.* qPCR on clinical FFPE samples suggestive for histoplasmosis or other invasive fungal infection with results of a broad-range fungal qPCR targeting the 28S ribosomal RNA gene (28S qPCR) to get insights into the best molecular strategy to identify histoplasmosis from FFPE samples.

## 2. Materials and Methods

### 2.1. DNA Extractions and qPCR Amplification Conditions

For DNA extraction from FFPE specimens, aliquots of four 5 µm sections were placed in Eppendorf Biopur^®^ tubes (Eppendorf AG, Hamburg, Germany). One extraction-negative control, consisting of DNA free water per three samples, was processed in parallel. The samples were deparaffinized using octane. DNA was extracted using the Master Pure Yeast DNA Purification Kit (Epicentre Biotechnologies, Madison, WI, USA) at 90 °C for 3 h with an additional bead-beating step to optimize fungal cell lysis. After a protein precipitation step, the DNA was precipitated by isopropanol, washed with 70% ethanol, and stored in 75 µL Triton X 0.1% at 4 °C for the short term (<7days) or at −20 °C for the longer term [25].

Each of these DNAs was studied by four different qPCRs. To document successful DNA extraction, a human DNA detecting qPCR [26] was used. An internal amplification control DNA (IAC qPCR) was used to determine PCR inhibitory activities. Fungal DNA was amplified using a broad-range 28S qPCR and a specific qPCR assay to detect *Histoplasma* DNA [23,26]. The extraction-negative controls were treated accordingly. All these qPCRs were performed on the 7500 Real-Time PCR System (Applied Biosystems, Foster City, CA, USA). Samples were tested in single tubes in the human DNA-detecting qPCR and in the IAC qPCR for 40 cycles or in duplicates in the 28S qPCR and the *H.* qPCR for 45 cycles. If inhibition (delta cycle threshold (CT) of more than 2 cycles) was detected and fungal qPCRs remained negative, DNA was again purified from the protein precipitation step of the extraction kit onwards in an attempt to remove the remaining PCR inhibitors [26].

The broad-range qPCR, targeting the ribosomal 28S rDNA genes (primer 10F and 12R), was performed as elsewhere described [21,27]. Each PCR run included six to eight no-template controls (NTC) and a standard curve ranging from 10 fg to 1 ng/reaction of *C. albicans* (ATCC 10231) DNA. A positive broad-range qPCR was defined as amplification of a fungal DNA in duplicates in less than 40 CTs with identical peaks in the melting curve analysis (±1 °C) in the absence of positive no-template, or extraction-negative controls. Amplicons of broad-range qPCR positive samples were sequenced. Amplicons were identified by BLAST search in GenBank [28]. Sequence identity of 98% and more was considered as the identification at genus level.

For the *H.* qPCR, targeting a region in the internal transcribed spacer 1 (ITS1) rDNA, the primers and the TaqMan probe were used as described by Simon et al. [23]. The Master Mix was replaced by TaqMan^®^ Universal PCR Master Mix II with uracil-N-glycosylase (Thermo Fisher, Schwerte, GE, Germany). Each run included four NTCs and a standard curve ranging from 10 fg to 1 ng/reaction of *Histoplasma* DNA. In contrast to the original publication, each sample which attained any positive result in the absence of positive NTCs and negative extraction controls was considered as positive.

### 2.2. Analytic Sensitivity and Specificity of the Histoplasma-Specific qPCR

For the determination of the analytical sensitivity and specificity, one clinical *Histoplasma* isolate (RKI 12-0644), nineteen other fungal isolates, and a human DNA preparation (Roche Applied Sciences; Cat. No. 11691112001, Indianapolis, IN, USA) were used (Table 1). Fungal strains were selected considering the most important fungal pathogens and colonizers in humans. In addition, we included fungi potentially mimicking histoplasmosis in histological samples.

For the calibration curve, the *Histoplasma* DNA was standardized at 1 ng/reaction and then serially diluted 10-fold to obtain solutions ranging from 1 ng to 1 fg/reaction. The sensitivity threshold was analyzed by running these DNA solutions on every *H.* qPCR run (in duplicates for the 1 fg/reaction standard). The analytic sensitivity (LOD) was defined as the lowest concentration which could be detected with a reasonable certainty (95%) [29]. Every positive test, even if only one of the duplicates were positive, was interpreted as a positive result.

In order to screen for cross-amplification of nontarget DNA, DNA of other fungi and human DNA were first standardized at 100 pg/reaction by the Qubit fluorometer using the dsDNA HS Assay Kit (Thermo Fisher, Schwerte, GE, Germany) and by the 28S qPCR, and then tested in duplicates in the *H.* qPCR. The identity of the strains was confirmed by sequencing the ITS region and PCR inhibition of the DNA standards was excluded by testing an internal amplification control (IAC) via qPCR. Inhibition was defined as a delta CT of more than 2 cycles.

### 2.3. Clinical Sensitivity and Specificity

FFPE tissue samples were cut into 5 µm thick sections. The first and the last sections were placed on slides and stained by Grocott’s methenamine silver stain (GMS) with Hematoxylin Eosin or Lightgreen counterstain. For each sample, these two slides were examined under the microscope for fungal elements.

**Figure 1:**
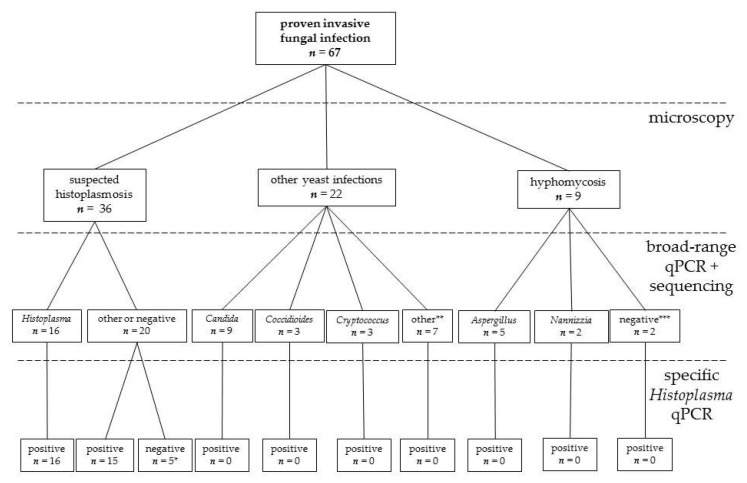
Formalin-fixed, paraffin-embedded tissue samples from patients with proven invasive fungal infections used for diagnostic accuracy testing of qPCR assays. Samples are classified according to the morphology of fungal elements by Grocott’s methenamine silver stain in tissue and results from sequencing of the amplicon of a broad-range qPCR. * Broad-range PCR suggested alternative etiologic agents in 3 suspected histoplasmosis samples (paracoccidioidomycosis: *n* = 2; emergomycosis: *n* = 1). Specific and broad-range PCR remained negative in two samples. ** Two were positive in the 28S qPCR, but sequencing showed mixed sequences. Histologically, both were suggestive for candidiasis. Five samples were negative in the 28S qPCR (histologically: blastomycosis (*n* = 1); coccidioidomycosis (*n* = 1), candidiasis (*n* = 3)). *** One was histologically a suspected aspergillosis and the other one a suspected dermatophytosis.

For the analysis of the clinical sensitivity and specificity [24], FFPE tissue samples from patients with histologically proven invasive fungal infection were used [30]. A total of 36 specimens (human *n* = 29 from 22 patients; animal *n* = 7 from 4 animals; one badger and three cats) suggestive for histoplasmosis defined by demonstration of small yeast cells (2–4 µm in size) with narrow-based budding, especially when grouped in clusters inside macrophages, were included (Table 2 and Figure 1) [17]. Subsequently, three of these samples were excluded from this analysis, as the 28S qPCR suggested an alternative diagnosis (samples 34–36).

In addition, 31 FFPE tissue specimens (human *n* = 29; animal *n* = 2) suggestive for other fungal infections by histopathology were included (Table 2 and Figure 1) to analyze the clinical specificity. If microscopy showed septated hyphae with acute angle branching, they were classified as hyphomycosis [17]. Broad-based budding yeasts up to 10–15 µm were considered suggestive for blastomycosis [17]. Histological evidence for cryptococcosis was defined as presence of narrow-based budding yeasts (4–10 µm) with a thick capsule [17]. Spherules (10 to 100 µm in size) with multiple endospores (2–5 µm) were defined as evidence for coccidioidomycosis [17]. For dermatophytosis, the presence of septated hyphae within the stratum corneum was considered as suggestive [17]. Candidiasis was considered when 3 to 5 µm in diameter yeasts were seen in the tissues, especially if intermingled with pseudohyphae [17].

### 2.4. Statistical Methods

Clinical sensitivity was defined as the percentage of positive results by the *H.* qPCR and by the 28S qPCR in the group of histologically suspected histoplasmosis with no alternative etiology detected by the 28S qPCR (*n* = 33) and clinical specificity was defined as the percentage of negative results for *Histoplasma* by the tested assays in the group of the other fungal infections, i.e., histopathology not suggestive for histoplasmosis (Table 2) [24,29]. Proportions in groups were compared by Chi-squared or Fisher’s exact test calculated in GraphPad Prism 7.04. A *p* < 0.05 in a two-sided test was considered statistically significant.

## 3. Results

### 3.1. Analytical Sensitivity and Specificity

The probability to detect 1 fg/reaction of *Histoplasma* DNA standard in the *H*. qPCR, at least in one sample of the duplicates, was 100%. Thus, 1 fg/reaction of *Histoplasma* DNA was defined as the analytical sensitivity. If only duplicates were considered as positive, as described in the original publication [23], the analytical sensitivity would have been 10 fg/reaction. This result is comparable with the results of the study of Simon et al. (analytical sensitivity = 50 fg of DNA per assay) [23]. The analytical specificity of the *H*. qPCR was 100%, as DNAs of other fungi were negative (Table 1).

### 3.2. Clinical Sensitivity and Specificity

**Figure 2:**
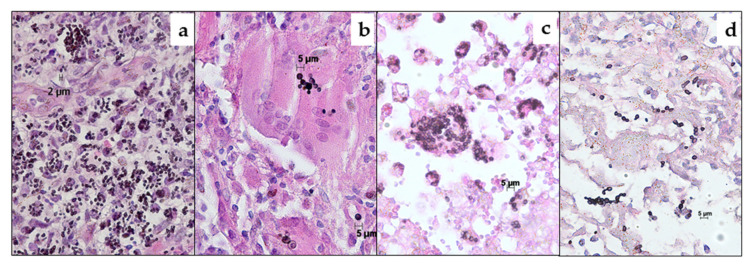
Tissue morphology of (**a**) emergomycosis (sample No. 36), (**b**) paracoccidioidomycosis (sample No. 35), (**c**) histoplasmosis (sample No. 2) and (**d**) *Candida glabrata* infection (sample No. 43) demonstrating all 2–4 µm budding yeast cells in clusters. Stained by Grocott’s methenamine silver (GMS) with Hematoxylin Eosin (HE) counterstain (×400).

No NTCs and extraction-negative controls were tested positive with the *H*. qPCR. Among the 36 histologically positive samples, seven samples (19%) were inhibited (animal origin: *n* = 4; human origin: *n* = 3). Despite PCR inhibition, two of these were positive in both fungal qPCRs without re-extraction (Nos. 4 and 16) (Table 2). Two were positive in the *H*. qPCR without re-extraction, and remained negative in the 28S qPCR (Nos. 27 and 28). Three inhibited samples negative in both qPCRs became positive in the *H*. qPCR and remained negative in the 28S qPCR after re-extraction (Nos. 19–21). The *H*. qPCR yielded positive results in 31 of 36 (86%) samples suggestive for histoplasmosis by histopathology. In one of the negative samples, DNA of *Emergomyces* sp. (No. 36) and in two samples DNA of *Paracoccidioides* sp. (Nos. 34, 35) were amplified by the 28S qPCR and identified by sequencing (Figure 2). These samples were excluded from the clinical sensitivity analysis, leading to a clinical sensitivity of 94% (31/33). The two remaining samples which were negative in the *H*. qPCR also yielded a negative result in the 28S qPCR.

The 28S qPCR showed a clinical sensitivity of 48.5% (16/33) for the diagnosis of histoplasmosis. Of the 33 *Histoplasma* samples, 17 had a negative 28S qPCR result for *Histoplasma* in the presence of a positive result in the *H*. qPCR. Three of these seventeen samples (i.e., 28S qPCR-negative but *H*. qPCR-positive samples) yielded a signal after 40 CTs in the *H*. qPCR, and of these, one showed a double-positive result (No. 30). This one belonged to a patient who was diagnosed also by culture. Two showed only a single positive result (Nos. 23 and 24) and belonged to another patient. Hence, the clinical sensitivity of the *H*. qPCR was significantly better than the clinical sensitivity of the 28S qPCR for diagnosing histoplasmosis from FFPE samples (31/33; 94% vs. 16/33; 48.5%; *p*-value < 0.0001).

Sequencing of the 28S qPCR amplicon in samples suggestive for histoplasmosis yielded a mixed sequence in one sample (No. 26), Saccharomyces cerevisiae (No. 30) and Candida spec. (No. 31) in another sample.

Of the 31 FFPE samples suggestive for other fungal infections by histopathology (candidiasis: *n* = 14, hyphomycosis: *n* = 6, coccidioidomycosis: *n* = 4, cryptococcosis: *n* = 3, dermatomycosis; *n* = 3, blastomycosis; *n* = 1), only two were inhibited (animal origin: *n* = 1; human origin: *n* = 1). One of these became positive in the 28S qPCR after re-extraction (No. 56), the other one remained negative (No. 67). All were negative in the *H*. qPCR. This leads to a diagnostic specificity of 100% for histoplasmosis.

## 4. Discussion

We confirm the excellent analytical sensitivity and specificity of this previously published specific qPCR targeting the ITS gene of *Histoplasma*. We demonstrated superior sensitivity of this assay compared to a 28S qPCR from pathology blocks. However, this 28S qPCR identified alternative fungal etiologies including the recently described emergomycosis. Therefore, the application of these PCR assays on tissue samples may be a successful approach to amplify fungal DNA from pathology blocks in order to identify the etiology of fungal infections suggestive for histoplasmosis by histopathology. This may improve the management of individual cases and provide an alternative approach to define endemic regions and the prevalence of histoplasmosis.

One explanation for the better performance of the specific qPCR could be a lower LOD of the *H*. qPCR due to the difference in size of the amplicons (63 base pairs for the *H*. qPCR vs. 339 ± 7 for the 28S qPCR), which has also been proposed for other fungal infections [31]. The beneficial effect of a shorter amplicon may be increased in FFPE samples in the context of DNA degradation by formaldehyde [32]. If there is a mixed infection or concomitant colonization by other fungi, and the amount of *Histoplasma* DNA is below the LOD in the 28S qPCR, this could be an explanation for the preferential amplification of the DNA present in larger amounts, as *Candida* or *Saccharomyces* (Nos. 30 and 31) in digestive samples.

There was no tissue sample suggestive for histoplasmosis which was tested negative in the *H*. qPCR but from which *Histoplasma* DNA could be amplified by the broad-range assay, suggesting that targeting the highly variable ITS region may not have impaired the sensitivity of this assay by variation in primer or probe binding sites. However, 28S qPCR was able to identify alternative fungal infections in three samples suggestive for histoplasmosis by histopathology (emergomycosis: *n* = 1; paracoccidioidomycosis: *n* = 2) which were tested negative in the *H*. qPCR. Explanations for negative results in the *H*. qPCR and in the 28S qPCR in two samples could be a DNA amount below the limit of detection of both qPCRs.

Histoplasmosis in non-endemic regions may be diagnosed when lesions of the lung or other organs are removed to exclude malignancy. Whether a positive *H*. qPCR in the presence of a histologically suspected histoplasmosis in this scenario is a sign for an active *Histoplasma* infection, or if a negative *H*. qPCR in the presence of a histologically suspected histoplasmosis is a sign for an inactive *Histoplasma* infection, remains unclear.

Limitations of this study include, first, that it is a retrospective study with a potential for selection bias, i.e., the clinical sensitivity of our assays may be lower in unselected samples. Second, the FFPE samples with other fungal infections had been selected on the basis of prior identification. This is the reason why we did not calculate an overall diagnostic sensitivity of the 28S qPCR. Third, only three samples (Nos. 19, 20, and 30) of our dataset were from a patient with a culturally proven *Histoplasma* infection.

There are only a limited number of publications about specific molecular diagnosis of *Histoplasma* from FFPE samples [33,34,35,36,37], and only two that examine the clinical specificity with *Histoplasma-*negative clinical FFPE samples [33,37]. A direct comparison of the analytical sensitivities of the different PCR assays described is difficult, as quantifications were based on plasmid DNAs, genomic DNAs, or on number of copies.

Our study of 67 FFPE samples is the largest examining both clinical sensitivity and clinical specificity of a specific *H.* qPCR applied on clinical FFPE samples. Previously, a study compared a nested PCR specific for *Histoplasma* (targeting the gene encoding a 100-kDa-like protein) with a broad-range PCR followed by sequencing. Herein, four histologically suspected histoplasmosis samples were analyzed. Of these, only one samples was positive for *Histoplasma* by broad-range fungal PCRs (one PCR targeting the 18S rRNA and a PCR targeting the ITS-2 region), and the same sample was also the only one positive in the specific *Histoplasma* nested PCR [37]. As in our study, the broad-range PCRs identified an alternative fungal agent (*Candida parapsilosis*) in a sample suggestive for histoplasmosis by histopathology [37]. Histoplasmosis is a fungal infection diagnosed in animals outside classically described endemic regions. In Europe, there have been several publications of *Histoplasma* infections in wild (badgers, hedgehogs) and domesticated animals (cats, horses, dogs) [38,39,40,41,42,43,44,45,46,47], which point toward a still-unknown reservoir in the environment of these animals. Furthermore, there have been two publications suggesting autochthonous human histoplasmosis infections in several European states such as Italy, Turkey, France, and Germany [48,49]. Whether histoplasmosis is an underestimated health issue in Europe remains unclear, as diagnostics are seldom asked in samples from patients without travel history.

In conclusion, using this specific *H.* qPCR on FFPE tissue samples helps to establish the diagnosis of histoplasmosis, guiding patient care. In a larger context, the use of this assay on tissue blocks may help to identify the prevalence of histoplasmosis in different regions, bringing further insights to endemic regions and guiding public health interventions. Depending on the pretest probability of histoplasmosis, the subsequent use in *H.* qPCR-negative samples or the additional use of this broad-range fungal assay could be beneficial to identify alternative fungal etiologies that might not be differentiated by histopathology, including emergomycosis, paracoccidioidomycosis and candidiasis.

## Figures and Tables

**Table 1:** Strains and human genomic DNA used to confirm the analytical sensitivity and specificity of the Histoplasma-specific qPCR.

Strains DNA Standardized at 100 pg/Reaction	Origin	*Histoplasma* qPCR Result
*Aspergillus fumigatus*	ATCC 46645	negative
*Blastomyces dermatitidis*	ATCC 18188	negative
*Blastomyces percursus*	CBS 139878	negative
*Candida albicans*	ATCC 10231	negative
*Candida glabrata*	ATCC 64677	negative
*Coccidioides immitis*	CBS 113856	negative
*Coccidioides posadasii*	CBS 113859	negative
*Cryptococcus gattii*	CBS 6289.85	negative
*Cryptococcus neoformans H99*	CBS 8710	negative
*Cunninghamella bertholletiae*	RKI 99-0536	negative
*Emergomyces africanus*	CBS 136260	negative
*Emergomyces europaeus*	RKI 17-1077	negative
*Emergomyces orientalis*	CBS 124587	negative
*Emergomyces pasteurianus*	CBS 101426	negative
*Emmonsia parva*	CBS 139881	negative
*Exophiala dermatitidis*	CBS 748.88	negative
*Histoplasma capsulatum*	RKI 12-0644	positive
*Paracoccidioides brasiliensis*	ATCC MYA-826	negative
*Talaromyces marneffei*	RKI 16-0774	negative
*Trichophyton violaceum*	RKI 16-0839	negative
Human Genomic DNA	Roche^®^ Cat. No. 11 691 112 001	negative

ATCC: American Type Culture Collection; CBS: Centraalbureau voor schimmelcultures (now known as Westerdijk Fungal Biodiversity Institute), Utrecht, Netherlands; RKI: strains isolated from fungal cultures derived from patient samples at the Robert Koch Institute.

**Table 2:** Formalin-fixed, paraffin-embedded tissue samples used for the determination of the clinical sensitivity and specificity of the qPCR assays.

No.	Organ	Fungal Etiology Suspected by Histopathology	28S Broad-Range qPCR with Subsequent Sequencing	*Histoplasma-*Specific qPCR
1 ^a^	skin	histoplasmosis	*Histoplasma capsulatum*	positive
2 ^a^	skin	histoplasmosis	*Histoplasma capsulatum*	positive
3 ^a^	mucosa	histoplasmosis	*Histoplasma capsulatum*	positive
4 ^a, b^	skin	histoplasmosis	*Histoplasma capsulatum*	positive
5	vocal fold	histoplasmosis	*Histoplasma capsulatum*	positive
6	lung	histoplasmosis	*Histoplasma capsulatum*	positive
7	lymph node	histoplasmosis	*Histoplasma capsulatum*	positive
8	adrenal gland	histoplasmosis	*Histoplasma capsulatum*	positive
9	esophagus	histoplasmosis	*Histoplasma capsulatum*	positive
10	duodenum	histoplasmosis	*Histoplasma capsulatum*	positive
11	lung	histoplasmosis	*Histoplasma capsulatum*	positive
12	lung	histoplasmosis	*Histoplasma capsulatum*	positive
13	lung	histoplasmosis	*Histoplasma capsulatum*	positive
14	intestine	histoplasmosis	*Histoplasma capsulatum*	positive
15	lung	histoplasmosis	*Histoplasma capsulatum*	positive
16 ^a, b^	skin	histoplasmosis	*Histoplasma capsulatum*	positive
17	lung	histoplasmosis	negative	positive
18	bone	histoplasmosis	negative	positive
19 ^b, c^	intestine	histoplasmosis	negative	positive
20 ^b, c^	intestine	histoplasmosis	negative	positive
21 ^b^	oral mucosa	histoplasmosis	negative	positive
22	lymph node	histoplasmosis	negative	positive
23	lung	histoplasmosis	negative	positive
24	lung	histoplasmosis	negative	positive
25	lung	histoplasmosis	negative	positive
26	lung	histoplasmosis	mixed sequence	positive
27 ^a, b^	skin	histoplasmosis	negative	positive
28 ^a, b^	skin	histoplasmosis	negative	positive
29 ^a^	skin	histoplasmosis	negative	positive
30 ^c^	intestine	histoplasmosis	*Saccharomyces cerevisiae*	positive
31	intestine	histoplasmosis	*Candida albicans*	positive
32	lung	histoplasmosis	negative	negative
33	lung	histoplasmosis	negative	negative
34	lung	histoplasmosis	*Paracoccidioides* spec.	negative
35	lung	histoplasmosis	*Paracoccidioides* spec.	negative
36	skin	histoplasmosis	*Emergomyces* spec.	negative
37	brain	blastomycosis	mixed sequence	negative
38	lung	coccidioidomycosis	*Coccidioides* spec.	negative
39	lung	coccidioidomycosis	*Coccidioides* spec.	negative
40	bone	coccidioidomycosis	*Coccidioides* spec.	negative
41	lung	coccidioidomycosis	negative	negative
42	unknown	candidiasis	*Candida glabrata*	negative
43	unknown	candidiasis	*Candida glabrata*	negative
44	unknown	candidiasis	*Candida glabrata*	negative
45	unknown	candidiasis	*Candida dubliniensis*	negative
46	unknown	candidiasis	*Candida dubliniensis*	negative
47	intestine	candidiasis	negative	negative
48	unknown	candidiasis	mixed sequence	negative
49	unknown	candidiasis	*Candida glabrata*	negative
50	paranasal sinus	candidiasis	*Candida albicans*	negative
51	unknown	candidiasis	*Candida albicans*	negative
52	unknown	candidiasis	mixed sequence	negative
53	unknown	candidiasis	negative	negative
54	unknown	candidiasis	negative	negative
55	paranasal sinus	candidiasis	negative	negative
56^b^	unknown	cryptococcosis	*Cryptococcus* spec.	negative
57	unknown	cryptococcosis	*Cryptococcus* spec.	negative
58	unknown	cryptococcosis	*Cryptococcus* spec.	negative
59	unknown	hyphomycosis	*Aspergillus fumigatus*	negative
60	unknown	hyphomycosis	*Aspergillus fumigatus*	negative
61	unknown	hyphomycosis	*Aspergillus fumigatus*	negative
62	unknown	hyphomycosis	*Aspergillus fumigatus*	negative
63	unknown	hyphomycosis	*Aspergillus fumigatus*	negative
64	unknown	hyphomycosis	negative	negative
65 ^a^	skin	dermatophytosis	*Nannizzia gypsea*	negative
66	skin	dermatophytosis	*Nannizzia gypsea*	negative
67 ^a, b^	skin	dermatophytosis	negative	negative

^a^ samples derived from animals, ^b^ inhibited samples, ^c^ samples from a patient with a culture-proven histoplasmosis.
